# Association between Perceived Activity Restriction Due to People’s Perception of Aging and Unmet Medical Needs among Middle-Aged and Elderly People: A Population-Based Study

**DOI:** 10.3390/medicina60010087

**Published:** 2024-01-02

**Authors:** Jeong-Min Yang, Min-Soo Kim, Ji-Sung Hong, Jae-Hyun Kim

**Affiliations:** 1 Department of Public Health, General Graduate School of Dankook University, Cheonan 31116, Republic of Korea; jeongmin1206@dankook.ac.kr (J.-M.Y.); soo931108@gmail.com (M.-S.K.); entlfch@dankook.ac.kr (J.-S.H.); 2 Institute for Health & Medical Policy, Dankook University, Cheonan 31116, Republic of Korea; 3 Department of Health Administration, College of Health Science, Dankook University, Cheonan 31116, Republic of Korea

**Keywords:** activity restriction, aging perception, unmet medical need, middle-aged and elderly people, KNHNES, chronic disease

## Abstract

*Background and Objectives*: The older members of a population might experience unmet medical needs, despite desiring medical care due to activity limitations driven by their perception of aging. This study conducted a cross-sectional analysis of the association between perceived activity restriction (PAR) due to people’s own perception of aging and unmet medical needs (UMN) in late middle-aged and older Koreans based on the Korean National Health and Nutrition Examination Survey (KNHANES). *Materials and Methods*: The 2016–2020 KNHANES was used to analyze a total of 2008 participants among groups aged 45 years or older by applying individual weights imposed from the raw data. The independent variable of PAR was assessed using self-reported questionnaires based on the global activity limitation indicator. Also, the dependent variable of UMN, referring to the state in which a patient’s medical care or service was insufficient, inadequate, or lacking, was assessed using a single question. After excluding missing values, the data on 2008 individuals were analyzed using a chi-square test, weighted logistic regression, and a stratified analysis of gender, age, and the presence of chronic illnesses. *Results*: The group that experienced PAR had an OR 2.13 higher (odds ratio [OR]: 2.13; 95% confidence interval [CI]: 1.27–3.56) to present UMN than the group that did not experience PAR. Furthermore, the results of the stratified analysis indicated that, in the group of female participants with chronic illness and in the group of elderly people, experiencing PAR was associated with a higher experience of UMN. *Conclusions*: There was a close association between PAR and UMN. In particular, when PAR occurred in the group of female participants with chronic illness and in the group of elderly people, the incidence rate of UMN was also found to be high. This finding highlights the need for policies and institutional measures to reduce UMN within vulnerable groups with an increased risk of medical inaccessibility due to activity restriction.

## 1. Introduction

In the 21st century, the key focal points within the health and medical sphere encompass low birthrates and aging. Notably, Korea stands out with a rapid progression in both low birth rates and an aging population, surpassing other nations [1]. As of October 2021, Korea’s fertility rate stands at 0.808, the lowest among United Nations (UN) member countries [2]. The aging population has also increased, with approximately 9.01 million individuals aged 65 years or older in Korea as of 2022, constituting 17.5% of the total population. By 2025, the country is projected to transition into a super-aged society [3]. This demographic shift towards an elderly population has given rise to an increasing concern about unmet medical needs (UMN), particularly among the elderly. In Korea, the UMN incidence rate among the elderly stands at 17.9%, approximately 5% higher than that in the general adult population [4]. Similarly, in European nations, the UMN incidence among the elderly is at 23.0%, with a noted escalation as they advance in age [5,6].

The dependent variable examined in this research, UMN, refers to instances when medical services deemed necessary by experts, such as healthcare professionals, are not accessed, even when desired by medical consumers [7]. In Korea, where rapid aging is underway, UMN triggers diverse physiological changes alongside swift alterations in the surrounding environment [8]. Particularly within the elderly demographic, UMN can disrupt timely treatment, escalating disease severity and fostering the progression of chronic ailments and mortality rates [9]. Prior studies indicate a correlation between UMN and the exacerbation of chronic diseases, as well as a decline in subjective health levels [5,10].

The burgeoning elderly population poses an additional societal challenge as the demand for both physical and mental support among seniors rises in tandem with aging [11]. According to the Korea Institute for Health and Social Affairs (KIHASA), 31.9% of individuals aged 65 years or older exhibit one or more disabilities in the activities of daily living (ADL) measure, signifying an increasing populace unable to live independently [11,12]. In a separate study, individuals experiencing perceived activity restriction (PAR) due to their own aging perceptions displayed heightened levels of depression compared to those without such experiences [13]. Furthermore, PAR due to people’s own aging perceptions is a problem that needs solving, in that the group which experienced PAR due to their own perception of aging had nearly four times more experience of UMN over a one year period than the group that had not experienced it. In addition, PAR has been found to be associated with stroke, hypertension, joint disease, lung disease, endocrine disease, and cancer [14]. Consequently, the prevalence of PAR, impacting quality of life and contributing to chronic illnesses, is anticipated to gradually increase. Indeed, as per the Korea National Health and Nutrition Examination Survey (KNHANES) data from 2017, the prevalence of PAR among individuals aged 65 years or older in Korea stood at 20.6%, which was higher than the 19.6% recorded in 2016. Studies in Japan, the Netherlands, and the United States project a gradual increase in these countries’ PAR rates [15,16,17,18].

The curtailment of activities linked to people’s own perception of aging poses a significant societal concern, notably among middle-aged and elderly individuals. Typically, this demographic plays pivotal roles, providing family support through employment [19] while also fostering networks within society, contributing to personal fulfillment and income generation, and sharing supervisory, educational, and technological responsibilities with younger generations [20]. However, experiencing PAR due to one’s own perception of aging can result in social exclusion or isolation, eroding social networks and intensifying feelings of loneliness [21]. Moreover, limitations in external activities and reduced social engagement can diminish social connections and opportunities for involvement, potentially escalating financial strains and emotional distress. Specifically, PAR linked to people’s own perception of aging may detrimentally affect the lives of middle-aged and elderly individuals [22], impacting early detection and treatment of illnesses if access to medical services diminishes. Recognizing that issues stemming from restricted medical service access due to PAR following people’s own perception of aging could yield greater societal and economic burdens than solely emotional effects like social exclusion, isolation, reduced networks, and loneliness means that addressing these concerns warrants a comprehensive societal approach.

This study underscores the noteworthy connection established by several previous research endeavors, demonstrating the correlation between PAR and UMN [10,23,24,25]. A previous study in Korea surveyed the UMN of the elderly using public health centers, showing that there was a significant difference in PAR among those with UMN. Additionally, the analysis of factors associated with distinct elderly groups in a previous study by Hwang, B.-D indicated a statistically significant variance, particularly among individuals aged 65–74. Notably, concerning economic factors, the incidence of UMN was 4.312 times higher than younger group [24].

With a focus on this relationship, this study aims to substantiate the link between PAR due to people’s own perception of aging and UMN. The primary objective is to prevent the potential degradation of medical accessibility resulting from PAR among the middle-aged and elderly demographic. Furthermore, this study aims to supply foundational data for health promotion endeavors.

## 2. Materials and Methods

### 2.1. Data Source and Study Participants

In this study, raw data from 2016–2020 were used among KNHANES data to investigate the relationship between PAR due to people’s own perception of aging and UMN. KNHANES is a major survey conducted to understand the overall health status of people in Korea, presenting the direction of future health policies to the state. Currently, the data from this survey are used in various studies to develop and supplement health policies, compare health levels in various countries, promote health, and prevent diseases. The subjects of the KNHANES survey employed in this study were 192 households from the 7th survey (2016), and 23 sample households were selected using a systematic sampling method among the appropriate households, excluding nursing homes, military and prison facilities, and foreign households. Within the sample household, all household members aged 1 or older who satisfied the appropriate member requirements were selected to be the subjects of the survey.

**Figure 1:**
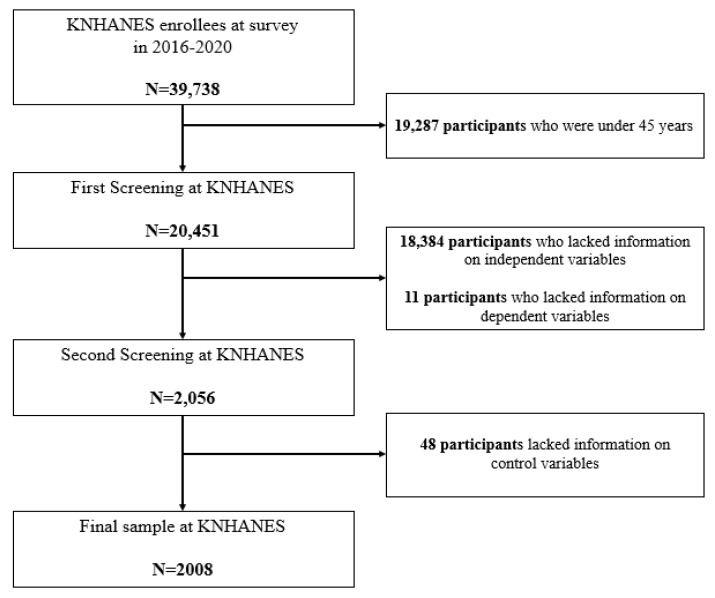
Flow chart for sample selection.

In this study, the relationship between PAR due to people’s own perception of aging and UMN was analyzed in a total of 2008 middle-aged and elderly people aged 45 or older, excluding those who had provided no information on the following characteristics: age, gender, residency region, education, marital status, self-rated health, occupation, health checkup, PAR, aerobic physical activity rate, smoking, drinking status, and prevalence of depression, the latter being a general characteristic of the KNHANES. Figure 1 depicts the flow chart for the sample selection process in this study.

### 2.2. Independent Variables

The independent variables included PAR due to one’s own perception of aging. PAR due to one’s own perception of aging was assessed by means of a Yes/No response to the following question: “Are you currently restricted to your activity due to mental health conditions or physical health conditions?”. The indicator based on the global activity limits indicator (GALI) was “Do you have a problem with your work (activities) because of your health condition?”. The responses were assigned to one of four subcategories: “very probable”, “probable”, “probably not”, and “not at all”. The GALI is a single-item survey instrument self-reported by the individual to assess health-related activity restrictions [26].

### 2.3. Dependent Variables

The dependent variable in this study was UMN. An unmet medical need was defined as ‘Have you ever needed a hospital treatment but not received it in the past 12 months?’. The measurement indicator for UMN utilized within Korea signifies whether individuals have experienced UMN, encompassing both outpatient and inpatient services in medical care [27].

### 2.4. Control Variables

In this study, predefined data from the community health survey such as “Gender”, “Marital status”, “Education level”, “Family income”, “Residency region”, “Occupation”, and “Health insurance type” were selected as the variables. The gender variable was divided into two categories: male and female. The age variable was divided into four categories: 45–54, 55–64, 65–74, and over 75 years of age. Marital status was divided into two categories: single (including separated and divorced) and married. The education variable was divided into four categories: under the elementary school level, middle school, high school, and over the college level. Family income was divided into three categories: poor, fair, and good. The residency region variable was divided into three categories: capital area, metropolitan city, and rural area. The occupation variable was divided into three categories: white-collar, blue-collar, and unemployment. Health insurance type was divided into three categories: national health insurance (regional), national health insurance (work), and medical benefits.

As variables for the health behavior factors, predefined data such as “Self-rated Health”, “Smoking status”, “Alcohol status”, and “Current Chronic Diseases” were selected as the variables. Self-rated health was categorized into three groups: poor, fair, and good. Smoking status was categorized into two groups: never and ever. Alcohol status was divided into two categories: never and ever. Finally, the current chronic disease variable (hypertension, dyslipidemia, stroke, cardiovascular disease, arthritis, osteoporosis, pulmonary disease, diabetes, cancer) was included as a covariate in our analyses.

### 2.5. Statistical Analysis

In this study, to analyze the association between PAR due to people’s own perception of aging and UMN, the age, gender, marital status, education, family income, residency region, occupation, health insurance type, self-rated health, current chronic disease, smoking status, alcohol status, and survey year variables of the study subjects were controlled.

In order to check the difference in the distribution of the dependent variable according to the independent variable, the Rao-Scott chi-square test was used for the ‘UMN’ dependent variable. In addition, a weighted logistic regression analysis was used to investigate the association between PAR due to people’s own perception of aging and UMN.

For all analyses, the criterion for statistical significance was *p* ≤ 0.05, two-tailed. All the analyses were carried out using the SAS statistical software package, version 9.4 (SAS Institute, Cary, NC, USA).

## 3. Results

### 3.1. General Characteristics of the Study Population

Table 1 shows the results of the general characteristics of the participants to determine the association between PAR due to people’s own perception of aging and unmet medical need. Of the 2008 participants, 19.4% (*n* = 389) experienced UMN. The group with PAR due to their own perception of aging accounted for 3.8% (*n* = 85) of the total subjects, while the UMN group was 32.4% of the total (*n* = 23). Among the control variables, the significant variables were gender, residency region, family income, health insurance type, self-rated health, and current chronic disease. In terms of gender, the rate of women experiencing UMN was 21.6%, which was higher than that of men, i.e., 16.2%. And, in the case of family income, the rate of having experienced unmet medical needs was highest among low-income families, at 23.7%.

### 3.2. Factors Associated with UMN in the Group of PAR Due to People’s Own Perception of Aging

Table 2 shows the results of the adjustment of the control variables to investigate the association between PAR due to people’s own perception of aging and UMN. Compared to the non-PAR group, the group with PAR due to their own perception of aging had higher odds of experiencing UMN, which was statistically significant (odds ratio [OR]: 2.13; 95% confidence interval [CI]: 1.27–3.56). Regarding sex, compared to the group of male participants, the group of female participants was associated with a 65% higher risk of experiencing UMN (OR: 1.65; 95% CI: 1.07–2.54).

### 3.3. Subgroup Analysis Stratified by Gender, Current Chronic Diseases, and Age

Table 3 shows the results of analyzing the association between PAR due people’s own perception of aging and unmet medical needs according to the gender and current chronic disease variables.

When the male participants experienced PAR due to their own perception of aging, their rate of experiencing UMN was 1.48 OR (OR: 1.48, 95% CI: 0.41~5.39), compared to the cases when there was no PAR, but it was not statistically significant. In the case of the female participants, their UMN experience rate was 2.21 OR (OR: 2.21, 95% CI: 1.23–4.00) when reporting PAR due to their own perception of aging compared to the cases with no PAR.

In the group without chronic disease, the UMN experience rate was 0.60 OR (OR: 0.60, 95% CI: 0.60–5.82) in the cases with PAR due to participants’ own perception of aging compared to the cases without PAR, but it was not statistically significant. In the case of the group with chronic diseases, the UMN experience rate was 2.25 OR (OR: 2.25, 95% CI: 1.30–3.89) when there was PAR, compared to when there was no activity limitation.

In the age-stratified analysis, for the middle-aged group, the prevalence of UMN experiences was 2.14 OR higher in the AR group compared to the non-AR group, although it did not reach statistical significance. Conversely, in the elderly group, the AR group exhibited a statistically significant 2.20 higher OR UMN prevalence compared to the non-AR group.

## 4. Discussion

In this study, we analyzed the relationship between PAR and UMN among individuals aged 45 years or older utilizing raw data from the 2016–2020 KNHANES. Using a logistic regression analysis, we conducted a detailed examination of the association with UMN, stratifying the PAR groups based on gender, age, and the presence or absence of chronic diseases.

The summary of the research results is as follows: The PAR group exhibited a UMN experience rate 2.13 times higher than the non-PAR group. Moreover, the female participants with PAR due to their own perception of aging had a higher UMN experience rate compared to the male participants. Additionally, the individuals with chronic diseases or those in the older age group demonstrated a robust association between PAR due to their own perception of aging and UMN when compared to the reference group.

The results of the study that the group experiencing PAR due to their own perception of aging had a higher UMN than the non-PAR group were consistent with previous studies [13]. According to previous studies, PAR groups have a higher rate of visits to outpatient medical facilities than non-PAR groups; meanwhile, in Korea, groups experiencing PAR due to their own perception of aging have less access to medical care than non-PAR groups [13,28]. The reason behind this phenomenon is that aging reduces physical function and increases the incidence of chronic diseases, increasing the need for medical care, but, unlike in other countries, in Korea it is difficult for activity-restricted people to engage in economic activities, which leads to a decrease in their income [4,29]. And, a decline in income leads to a decline in the use of medical facilities and services. In addition, in a previous study conducted on 1233 people aged 65 years or older in Korea, the rate of UMN experiences was four times higher in the group reporting PAR due to aging and dementia than in the group without PAR, and most of the reasons cited for the UMN experiences were ‘economic burden’ and ‘traffic inconvenience’ [13]. Therefore, in order to resolve PAR due to people’s own perception of aging, it is necessary to promote positive aging by encouraging participation in activities such as lifelong education and economic, leisure, and volunteer activities at the national level [30].

In addition, in this study, the UMN experience rate was higher in the female participants’ group when there were reports of PAR due to people’s own perception of aging compared to the male participants’ group. These results can be explained for several reasons. In a previous study involving 579 elderly people aged 65 years or older, female participants were found to have a higher rate of being unmarried, a lower education rate, a higher number of basic livelihood recipients, and less monthly income than middle-aged or male participants [31]. Furthermore, the analysis in question revealed that the female participants were more exposed to various vulnerabilities such as the burden of maintaining family balance and experiencing career discontinuity compared to the male participants [32]. It has already been established that PAR leads to a decrease in income, resulting in a higher UMN ratio due to economic burdens [4,13,29]. Lastly, considering previous studies which show that female participants experience more PAR than male participants, it is thought that the matter of women experiencing PAR due to their own perception of aging needs to be discussed from various perspectives [33,34]. To address this issue, policies are required to ease social restrictions and support women’s participation in economic activities.

Meanwhile, the findings of this study align with prior research, revealing a robust correlation between PAR due to people’s own perception of aging and UMN, particularly in groups with chronic diseases [35,36]. According to previous studies, the occurrence of chronic diseases in individuals with PAR exacerbates health issues, leading to intensified PAR and subsequent unmet medical needs [36]. Additionally, the reduction in participation in leisure activities, a crucial factor in enhancing the lives of those with chronic diseases, is associated with PAR, with people suffering from chronic diseases experiencing a heightened sense of PAR compared to those without such conditions [14]. Through these studies, it is interpreted that PAR due to people’s own perception of aging not only diminishes these people’s quality of life but also hinders positive aging by contributing to their unmet medical needs. To address this, it is essential to alleviate mobility challenges by providing transportation services. Simultaneously, local resources and support systems should be implemented to aid those people experiencing PAR due to their own perception of aging [36].

Finally, in the elderly group, it can be inferred that their unmet medical needs (UMN) are higher due to PAR itself rather than age perception-induced activity limitations. A prior study analyzing activity restriction and healthcare utilization among 754 individuals aged 70 years and older in the United States revealed that the group with restricted activity showed an inclination for increased healthcare services utilization due to illnesses. However, it was reported that, over the long term, their utilization of healthcare services decreased due to physical limitations [37]. Furthermore, according to a previous study investigating activity restriction and the incidence of UMN in Korea [38], it was reported that, as age increased, both the rate of activity restriction and the incidence of UMN increased. Also, in the above-mentioned study, the occurrence rate of UMN in the group with restricted activity was approximately 5% higher than that in the group not experiencing said restriction.

PAR due to people’s own perception of aging has been demonstrated as significantly threatening in prior studies. Moreover, the reason why we need to pay more attention to PAR due to people’s own perception of aging is that subjective health status, a factor which can assess this, is influenced by psychological and social aspects beyond physical health [38]. Studies conducted abroad have already utilized both subjective and objective measurement methods to identify PAR, including the use of the symptoms-response ratio (SSR) as the unmet medical needs (UMN) rate across specific diseases, medical services, employment sectors, and medical insurance types [39]. However, in Korea, most measurement methods are still primarily limited to surveys, and basic research aimed at reducing UMN in the group of people experiencing PAR has not been undertaken [39]. Therefore, it is anticipated that the rate of people with PAR experiencing UMN within the Korean healthcare system can be mitigated through the involvement of care providers such as families and long-term caregivers or by implementing various health checkups or healthcare methods [40]. Additionally, this study aims to provide foundational data for policy and institutional measures aimed at reducing UMN experienced among people with PAR due to their own perception of aging.

The limitations of this study are as follows: First, the study conducted a cross-sectional analysis using data from the 2016–2020 KNHNES, meaning that it is not possible to identify the causal relationship between PAR due to people’s own perception of aging and UMN. Second, as described above, there is a limitation in that PAR due to people’s own perception of aging is entirely judged by means of subjective evaluations, which are affected by psychological and social areas beyond physical health. Third, since the activity-limiting factor was only measured to be “aging”, it is not possible to accurately determine which area of limitation is caused by it. Despite these limitations, this study has the following strengths. Most studies have focused only on the physical limitations of AR due to people’s own perception of aging, but this study called for a detailed evaluation that focused on psychological and social areas beyond physical health. Furthermore, conducting research on the middle-aged and elderly population enabled the formulation of policies tailored to South Korea’s rapid aging trend.

## 5. Conclusions

This study examined the relationship between PAR due to aging and unmet medical needs among the middle-aged and elderly population. The group experiencing restricted activity due to aging showed a higher incidence of unmet medical needs, especially among women and those with chronic conditions. To address this, there is a necessity for social initiatives aimed at alleviating constraints faced by vulnerable groups, such as women who are prone to experiencing unmet medical needs and individuals managing chronic conditions requiring ongoing care. A multifaceted approach involving increased societal attention and healthcare system strategies is essential to reducing unmet healthcare needs. Considering that perceptions of old age impacting restricted activity encompass physical, psychological, and social dimensions, comprehensive research on this subject and health policies are imperative.

## Figures and Tables

**Table 1:** General characteristics of the study population with PAR due to their own perception of aging.

Variables	Total		UMN				*p*-Value
			No		Yes		
	N	% *	N	% *	N	% *	
Total	2008	100.0	1619	80.6	389	19.4	
PAR due to people’s own perception of aging							0.0071
No	1923	96.2	1557	81.1	366	18.9	
Yes	85	3.8	62	67.6	23	32.4	
Age							0.5425
45–54	237	19.9	190	79.9	47	20.1	
55–64	476	26.5	374	78.4	102	21.6	
65–75	667	26.0	542	82.5	125	17.5	
Over 75	628	27.6	513	81.5	115	18.5	
Gender							0.0133
Male	770	41.4	655	83.8	115	16.2	
Female	1238	58.6	964	78.4	274	21.6	
Marital Status							<0.0001
Single (including separated and divorced)	789	37.4	590	75.1	199	24.9	
Married	1219	62.6	1029	83.9	190	16.1	
Education							0.4443
≤Elementary school	1098	47.4	872	78.9	226	21.1	
Middle school	316	16.9	254	83.0	62	17.0	
High school	400	23.3	331	82.4	69	17.6	
≥College	194	12.4	162	80.7	32	19.3	
Family Income							0.0009
Poor	1022	47.8	775	76.3	247	23.7	
Fair	757	38.8	651	85.4	106	14.6	
Good	229	13.4	193	82.4	36	17.6	
Residency Region							0.4761
Capital area	434	24.6	339	78.4	95	21.6	
Metropolitan city	614	31.1	500	80.9	114	19.1	
Rural area	960	44.3	780	81.7	180	18.3	
Occupation							0.4923
White-collar	305	16.9	256	82.3	49	17.7	
Blue-collar	1584	77.9	1261	80.0	323	20.0	
Unemployment	119	5.2	102	84.9	17	15.1	
Health Insurance Type							0.0190
National health insurance (regional)	644	31.8	513	79.7	131	20.3	
National health insurance (work)	1064	53.3	884	82.9	180	17.1	
Medical benefits	300	14.9	222	74.5	78	25.5	
Self-Rated Health							<0.0001
Poor	1261	61.0	975	77.1	286	22.9	
Fair	637	33.0	545	85.1	92	14.9	
Good	110	6.0	99	91.5	11	8.5	
Current Chronic Disease ^†^							0.0220
No	250	14.7	218	86.9	32	13.1	
Yes	1758	85.3	1401	79.5	357	20.5	
Smoking Status							0.6474
Ever	1254	59.6	1002	80.2	252	19.8	
Never	754	40.4	617	81.2	137	18.8	
Alcohol Status							0.6135
Ever	489	22.2	396	81.5	93	18.5	
Never	1519	77.8	1223	80.4	296	19.6	

^†^ Hypertension, dyslipidemia, stroke, myocardial infarction, angina pectoris, osteoarthritis, osteoporosis, tuberculosis, asthma, diabetes, cancer/%. * Weighted percentage/PAR—perceived activity restriction/UMN—unmet medical needs.

**Table 2:** Factors associated with unmet medical needs in the group of people with PAR due to their own perception of aging.

Variables	UMN		
	Adjusted OR	95% CI	*p*-Value
PAR due to people’s own perception of aging			
No	1.00		
Yes	2.13	(1.27–3.56)	0.004
Age			
45–54	1.00		
55–64	1.00	(0.61–1.62)	0.984
65–75	0.65	(0.39–1.10)	0.107
Over 75	0.56	(0.32–0.96)	0.034
Gender			
Male	1.00		
Female	1.65	(1.07–2.54)	0.022
Marital Status			
Single (including separated and divorced)	1.00	-	
Married	1.42	(1.06–1.90)	0.018
Education			
≤Elementary school	0.93	(0.46–1.87)	0.828
Middle school	0.76	(0.37–1.55)	0.450
High school	0.79	(0.43–1.45)	0.443
≥College	1.00	-	
Family Income			
Poor	1.31	(0.80–2.16)	0.288
Fair	0.76	(0.45–1.27)	0.293
Good	1.00	-	
Residency Region			
Capital area	1.00	-	
Metropolitan city	0.89	(0.61–1.30)	0.546
Rural area	0.83	(0.60–1.15)	0.260
Occupation			
White-collar	1.00	-	
Blue-collar	1.02	(0.59–1.77)	0.947
Unemployment	0.67	(0.26–1.72)	0.406
Health Insurance Type			
National health insurance (regional)	1.00	-	
National health insurance (work)	0.89	(0.67–1.18)	0.406
Medical benefits	0.86	(0.55–1.34)	0.498
Self-Rated Health			
Poor	2.90	(1.42–5.95)	0.004
Fair	1.91	(0.90–4.04)	0.089
Good	1.00	-	
Current Chronic Disease ^†^			
No	1.00	-	
Yes	1.45	(0.90–2.35)	0.130
Smoking Status			
Ever	1.00	-	
Never	1.29	(0.84–1.99)	0.244
Alcohol Status			
Ever	1.00	-	
Never	1.13	(0.82–1.57)	0.449

^†^ Hypertension, dyslipidemia, stroke, myocardial infarction, angina pectoris, osteoarthritis, osteoporosis, tuberculosis, asthma, diabetes, cancer/PAR—perceived activity restriction/UMN—unmet medical needs.

**Table 3:** Factors associated with unmet medical needs in the group of people with PAR due to their own perception of aging, stratified according to gender and the existence of chronic diseases.

Variables	UMN			
	Male		Female	
	Adjusted OR	95% CI	Adjusted OR	95% CI
PAR due to people’s own perception of aging				
No	1.00		1.00	
Yes	1.48	(0.41–5.39)	2.21	(1.23–4.00) ***
**Variables**	**UMN**			
	**No Current Chronic Disease**		**Current Chronic Disease**	
	**Adjusted OR**	**95% CI**	**Adjusted OR**	**95% CI**
PAR due to aging perception				
No	1.00		1.00	
Yes	0.60	(0.60–5.82)	2.25	(1.30–3.89) ***
**Variables**	**UMN**			
	**Middle-aged group**		**Elderly group**	
	**Adjusted OR**	**95% CI**	**Adjusted OR**	**95% CI**
PAR due to aging perception				
No	1.00		1.00	
Yes	2.14	(0.89–5.11)	2.20	(1.31–3.69) **

**p < 0.01. *** *p* < 0.001. All covariates were controlled. PAR: perceived activity restriction; UMN: unmet medical needs.

## Data Availability

Data are owned by and are available from the database of the Korea National Health and Nutrition Examination Surveys (KNHNES) https://knhanes.kdca.go.kr/knhanes/main.do. KNHNES allows all of these data freely for any researcher who promises to follow the research ethics.
